# Bis[2-(1*H*-benzimidazol-2-yl)­phenolato]­dimethanol­manganese(III) chloride

**DOI:** 10.1107/S1600536810012894

**Published:** 2010-04-14

**Authors:** Qi Ma, Miaoli Zhu, Sisi Feng, Liping Lu

**Affiliations:** b College of Chemistry and Chemical Engineering, Shanxi Datong University, Datong, Shanxi 037009, People’s Republic of China; a Institute of Molecular Science, Key Laboratory of Chemical Biology and Molecular Engineering of the Education Ministry, Shanxi University, Taiyuan, Shanxi 030006, People’s Republic of China

## Abstract

In the title compound, [Mn(C_13_H_9_N_2_O)_2_(CH_3_OH)_2_]Cl, the Mn^III^ atom (site symmetry 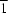) is coordinated by two *N*,*O*-bidentate 2-(1*H*-benzimidazol-2-yl)phenolate ligands and two methanol mol­ecules, to generate a distorted *trans*-MnN_2_O_4_ octa­hedral geometry for the metal ion. The dihedral angle between the aromatic ring systems in the ligand is 16.0 (3)°. In the crystal structure, the complex cations and chloride anions are linked by O—H⋯Cl and N—H⋯Cl hydrogen bonds. The chloride ion lies on a crystallographic twofold axis.

## Related literature

For our previous work on manganese complexes, see: Li *et al.* (2000 ▶, 2002 ▶).
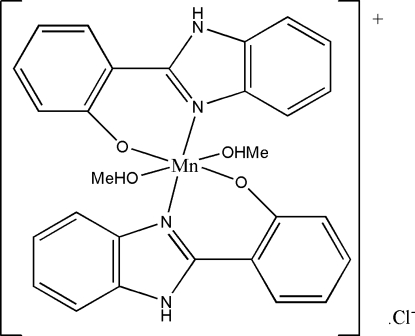


## Experimental

### 

#### Crystal data

[Mn(C_13_H_9_N_2_O)_2_(CH_4_O)_2_]Cl
*M* *_r_* = 572.92
Monoclinic, 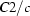

*a* = 17.897 (4) Å
*b* = 9.0349 (19) Å
*c* = 16.024 (3) Å
β = 93.502 (4)°
*V* = 2586.2 (10) Å^3^
*Z* = 4
Mo *K*α radiation
μ = 0.66 mm^−1^
*T* = 298 K
0.30 × 0.10 × 0.06 mm

#### Data collection

Bruker SMART 1K CCD diffractometer
Absorption correction: multi-scan (*SADABS*; Bruker, 2000 ▶) *T*_min_ = 0.827, *T*_max_ = 0.962
5075 measured reflections
2241 independent reflections
1444 reflections with *I* > 2σ(*I*)
*R*_int_ = 0.073

#### Refinement

*R*[*F* ^2^ > 2σ(*F* ^2^)] = 0.069
*wR*(*F* ^2^) = 0.195
*S* = 1.01
2241 reflections
179 parameters
1 restraint
H-atom parameters constrained
Δρ_max_ = 0.72 e Å^−3^
Δρ_min_ = −0.41 e Å^−3^

### 

Data collection: *SMART* (Bruker, 2000 ▶); cell refinement: *SAINT* (Bruker, 2000 ▶); data reduction: *SAINT*; program(s) used to solve structure: *SHELXS97* (Sheldrick, 2008 ▶); program(s) used to refine structure: *SHELXL97* (Sheldrick, 2008 ▶); molecular graphics: *SHELXTL/PC* (Sheldrick, 2008 ▶); software used to prepare material for publication: *SHELXTL/PC*.

## Supplementary Material

Crystal structure: contains datablocks I, global. DOI: 10.1107/S1600536810012894/hb5394sup1.cif

Structure factors: contains datablocks I. DOI: 10.1107/S1600536810012894/hb5394Isup2.hkl

Additional supplementary materials:  crystallographic information; 3D view; checkCIF report

## Figures and Tables

**Table 1:** Selected bond lengths (Å)

Mn1—O1	1.864 (4)
Mn1—N1	2.041 (4)
Mn1—O2	2.252 (4)

**Table 2:** Hydrogen-bond geometry (Å, °)

*D*—H⋯*A*	*D*—H	H⋯*A*	*D*⋯*A*	*D*—H⋯*A*
O2—H2O⋯Cl1^i^	0.88	2.25	3.107 (3)	165
N2—H2⋯Cl1	0.86	2.36	3.177 (5)	159

Symmetry code: (i) 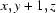.

**Fig. 1.**
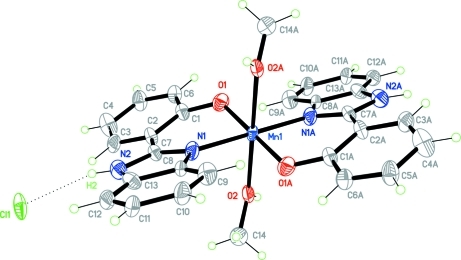
The structure of (I), with displacement ellipsoids drawn at the 30% probability level for non-H atoms. (Symmetry code: A 1 - x, 2 - y, -z.)

**Fig. 2.**
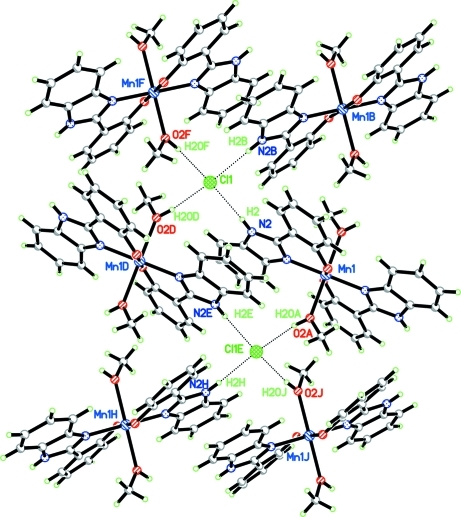
The H-bond network of (I). (Symmetry codes: A 1 - x, 2 - y, -z; B 1 - x, y, -z + 1/2; D x, y - 1, z; E 1 - x, 1 - y, -z; F 1 - x, y - 1, -z + 1/2; H x, 1 - y, z - 1/2; J x, 2 - y, z - 1/2.)

## supplementary crystallographic information

### Comment

As part of the ongoing study of manganese complexes (Li *et al.*, 2000, 2002), we now report the crystal structure of the title compound (I).

The geometric parameters of (I) are listed in Table 1. The molecular conformation is illustrated in Fig. 1. This compound consists of a Mn(III) 2-(1*H*-benzimidazol-2-yl)phenol complex cation and a chloride anion. The complex cation has a slightly elongated octahedral coordination of the metal ion through the formation of two Mn—N and four Mn—O bonds with two asymmetric bidentate 2-(1*H*-benzimidazol-2-yl)phenol ligands and two methanol molecules. The equatorial plane is formed by O1, O1A, N1, N1A from two ligands with the Mn—N bonds of 2.041 (4) Å and Mn—O bonds of 1.864 (4) Å respectively, similar to those in the related [Mn(C_13_H_8_N_2_OBr)_2_(C_5_H_5_N)_2_.3(C_5_H_5_N) (Li *et al.*, 2002), [Mn(C_20_H_14_N_2_O_2_)(H_2_O)(CH_3_OH)]ClO_4_ and [Mn(C_20_H_14_N_2_O_2_)(C_7_H_5_O_2_)].CH_3_CN Mn(III) compounds (Li *et al.*, 2000). The axial positions are occupied by two methanol O atoms with Mn—O distance of 2.252 (4) Å, slightly shorter than the axial Mn—O bonds [2.297 (5) and 2.287 (5) Å] in [Mn(C_20_H_14_N_2_O_2_)(H_2_O)(CH_3_OH)]ClO_4_ compound. Therefore, this complex exhibits a typically axial Jahn–Teller distortion characteristic of Mn(III) ions. The metal Mn atom is on a crystallographic inversion center. The dihedral angle between the benzimidazol group plane and the phenol group plane of the ligand is 16.0 (3)°, possibly resulting from the request of the coordination between Mn(III) and the ligands.

The hydrogen-bonding geometry in (I) is listed in Table 2 and illustrated as Fig. 2. There are two types of hydrogen bonds O—H···Cl and N—H···Cl in the crystal packing. Every chloride anion is involved in four such hydrogen bonds and lies on a two-fold axis.

### Experimental

2-(1*H*-benzimidazol-2-yl)phenol was synthesized by adding 20 ml of salicylaldehyde (20 mmol) ethanol solution to 60 ml of *O*-phenylenediamine (20 mmol) ethanol solution at 373 K and refluxing for an hour. The solvent was evaporated and the light yellow precipitate collected.

2-(1*H*-benzimidazol-2-yl)phenol (0.2 mmol) was disolved in 8 ml of methanol and MnCl_2_ (0.1 mmol) in 2 ml of water. Mixing the two solutions and the solution turn to black immediately. Stirring the solution for 10 minutes at room temperature. Filtered, the filtrate was left at room temperature and black slabs of (I) appeared from the solution after three days, by slow evaporation of the mixing solvent.

### Refinement

H atoms attached to C and N atoms of (I) were placed in geometrically idealized positions, with Csp^2^—H = 0.93, Csp^3^—H = 0.96 and Nsp^2^—H = 0.86 Å and constrained to ride on their parent atoms, with U_iso_(H) = 1.2U_eq_(Csp^2^ and Nsp^2^) or 1.5U_eq_(Csp^3^). H atom attached to O atom was located from difference Fourier map and refined its global U_iso_ value. The O—H distance is 0.877 Å.

### Crystal data

**Table:**

[Mn(C_13_H_9_N_2_O)_2_(CH_4_O)_2_]Cl	*F*(000) = 1184
*M**_r_* = 572.92	*D*_x_ = 1.471 Mg m^−^^3^
Monoclinic, *C*2/*c*	Mo *K*α radiation, λ = 0.71073 Å
Hall symbol: -C 2yc	Cell parameters from 1038 reflections
*a* = 17.897 (4) Å	θ = 2.3–21.8°
*b* = 9.0349 (19) Å	µ = 0.66 mm^−^^1^
*c* = 16.024 (3) Å	*T* = 298 K
β = 93.502 (4)°	Sheet, black
*V* = 2586.2 (10) Å^3^	0.30 × 0.10 × 0.06 mm
*Z* = 4	

### Data collection

**Table:**

Bruker SMART 1K CCD diffractometer	2241 independent reflections
Radiation source: fine-focus sealed tube	1444 reflections with *I* > 2σ(*I*)
graphite	*R*_int_ = 0.073
ω scans	θ_max_ = 25.0°, θ_min_ = 2.3°
Absorption correction: multi-scan (*SADABS*; Bruker, 2000)	*h* = −21→20
*T*_min_ = 0.827, *T*_max_ = 0.962	*k* = −10→10
5075 measured reflections	*l* = −17→19

### Refinement

**Table:**

Refinement on *F*^2^	Primary atom site location: structure-invariant direct methods
Least-squares matrix: full	Secondary atom site location: difference Fourier map
*R*[*F*^2^ > 2σ(*F*^2^)] = 0.069	Hydrogen site location: inferred from neighbouring sites
*wR*(*F*^2^) = 0.195	H-atom parameters constrained
*S* = 1.01	*w* = 1/[σ^2^(*F*_o_^2^) + (0.0997*P*)^2^] where *P* = (*F*_o_^2^ + 2*F*_c_^2^)/3
2241 reflections	(Δ/σ)_max_ < 0.001
179 parameters	Δρ_max_ = 0.72 e Å^−^^3^
1 restraint	Δρ_min_ = −0.41 e Å^−^^3^

### Special details

**Table:**

Geometry. All esds (except the esd in the dihedral angle between two l.s. planes) are estimated using the full covariance matrix. The cell esds are taken into account individually in the estimation of esds in distances, angles and torsion angles; correlations between esds in cell parameters are only used when they are defined by crystal symmetry. An approximate (isotropic) treatment of cell esds is used for estimating esds involving l.s. planes.
Refinement. Refinement of *F*^2^ against ALL reflections. The weighted *R*-factor wR and goodness of fit *S* are based on *F*^2^, conventional *R*-factors *R* are based on *F*, with *F* set to zero for negative *F*^2^. The threshold expression of *F*^2^ > σ(*F*^2^) is used only for calculating *R*-factors(gt) etc. and is not relevant to the choice of reflections for refinement. *R*-factors based on *F*^2^ are statistically about twice as large as those based on *F*, and *R*- factors based on ALL data will be even larger.

### Fractional atomic coordinates and isotropic or equivalent isotropic displacement parameters (Å^2^)

**Table:**

	*x*	*y*	*z*	*U*_iso_*/*U*_eq_	
Mn1	0.5000	1.0000	0.0000	0.0291 (4)	
N1	0.4766 (3)	0.7876 (5)	0.0331 (3)	0.0435 (12)	
N2	0.4849 (3)	0.5839 (5)	0.1099 (3)	0.0518 (14)	
H2	0.5016	0.5225	0.1476	0.062*	
O1	0.5985 (2)	0.9683 (4)	0.0406 (3)	0.0473 (11)	
O2	0.4667 (2)	1.0747 (4)	0.1266 (2)	0.0499 (11)	
C1	0.6238 (3)	0.8846 (6)	0.1061 (3)	0.0414 (14)	
C2	0.5854 (3)	0.7679 (6)	0.1370 (3)	0.0409 (14)	
C3	0.6160 (4)	0.6872 (6)	0.2031 (4)	0.0509 (16)	
H3	0.5890	0.6092	0.2244	0.061*	
C4	0.6883 (4)	0.7211 (7)	0.2396 (4)	0.065 (2)	
H4	0.7094	0.6676	0.2847	0.078*	
C5	0.7259 (3)	0.8380 (7)	0.2046 (4)	0.0541 (17)	
H5	0.7736	0.8635	0.2262	0.065*	
C6	0.6942 (3)	0.9148 (6)	0.1399 (4)	0.0472 (15)	
H6	0.7211	0.9914	0.1173	0.057*	
C7	0.5144 (3)	0.7165 (5)	0.0934 (3)	0.0327 (13)	
C8	0.4166 (3)	0.6958 (6)	0.0065 (4)	0.0436 (15)	
C9	0.3598 (4)	0.7060 (6)	−0.0577 (4)	0.0519 (17)	
H9	0.3553	0.7887	−0.0922	0.062*	
C10	0.3117 (3)	0.5900 (7)	−0.0673 (4)	0.0537 (17)	
H10	0.2731	0.5950	−0.1087	0.064*	
C11	0.3180 (4)	0.4667 (7)	−0.0184 (4)	0.0593 (19)	
H11	0.2836	0.3905	−0.0277	0.071*	
C12	0.3722 (4)	0.4514 (6)	0.0427 (4)	0.0537 (17)	
H12	0.3756	0.3669	0.0758	0.064*	
C13	0.4212 (3)	0.5632 (6)	0.0540 (3)	0.0411 (14)	
C14	0.4002 (4)	1.0293 (7)	0.1622 (4)	0.066 (2)	
H14A	0.3714	0.9686	0.1230	0.100*	
H14B	0.3715	1.1149	0.1756	0.100*	
H14C	0.4125	0.9737	0.2122	0.100*	
Cl1	0.5000	0.3350 (2)	0.2500	0.0652 (8)	
H2O	0.4843	1.1487	0.1572	0.031 (14)*	

### Atomic displacement parameters (Å^2^)

**Table:**

	*U*^11^	*U*^22^	*U*^33^	*U*^12^	*U*^13^	*U*^23^
Mn1	0.0406 (7)	0.0171 (5)	0.0290 (7)	−0.0039 (5)	−0.0035 (5)	0.0035 (5)
N1	0.061 (3)	0.026 (2)	0.043 (3)	−0.006 (2)	0.003 (2)	0.001 (2)
N2	0.080 (4)	0.030 (3)	0.047 (3)	0.003 (3)	0.014 (3)	0.007 (2)
O1	0.052 (3)	0.041 (2)	0.048 (3)	−0.0053 (18)	−0.0010 (19)	0.0075 (19)
O2	0.073 (3)	0.038 (2)	0.040 (2)	−0.017 (2)	0.011 (2)	−0.016 (2)
C1	0.054 (4)	0.035 (3)	0.035 (3)	0.002 (3)	−0.001 (3)	−0.006 (3)
C2	0.052 (4)	0.035 (3)	0.035 (3)	0.005 (3)	−0.003 (3)	−0.011 (3)
C3	0.071 (4)	0.023 (3)	0.059 (4)	−0.005 (3)	0.008 (3)	0.002 (3)
C4	0.101 (6)	0.046 (4)	0.045 (4)	0.029 (4)	−0.020 (4)	0.005 (3)
C5	0.048 (4)	0.047 (4)	0.065 (4)	0.007 (3)	−0.018 (3)	−0.013 (3)
C6	0.053 (4)	0.037 (3)	0.051 (4)	0.000 (3)	−0.002 (3)	−0.003 (3)
C7	0.051 (3)	0.023 (3)	0.024 (3)	0.008 (2)	0.001 (2)	0.004 (2)
C8	0.050 (4)	0.037 (3)	0.045 (4)	−0.019 (3)	0.016 (3)	−0.018 (3)
C9	0.074 (5)	0.037 (3)	0.044 (4)	0.003 (3)	−0.003 (3)	0.010 (3)
C10	0.052 (4)	0.052 (4)	0.055 (4)	−0.011 (3)	−0.009 (3)	−0.013 (3)
C11	0.073 (5)	0.041 (4)	0.066 (5)	−0.022 (3)	0.017 (4)	−0.017 (3)
C12	0.081 (5)	0.027 (3)	0.056 (4)	−0.010 (3)	0.025 (4)	−0.001 (3)
C13	0.048 (4)	0.047 (3)	0.028 (3)	0.011 (3)	0.003 (3)	0.001 (3)
C14	0.078 (5)	0.059 (4)	0.064 (5)	−0.017 (4)	0.022 (4)	−0.009 (3)
Cl1	0.142 (2)	0.0221 (10)	0.0309 (12)	0.000	−0.0013 (13)	0.000

### Geometric parameters (Å, °)

**Table:**

Mn1—O1	1.864 (4)	C3—H3	0.9300
Mn1—O1^i^	1.864 (4)	C4—C5	1.389 (9)
Mn1—N1	2.041 (4)	C4—H4	0.9300
Mn1—N1^i^	2.041 (4)	C5—C6	1.343 (8)
Mn1—O2	2.252 (4)	C5—H5	0.9300
Mn1—O2^i^	2.252 (4)	C6—H6	0.9300
N1—C7	1.312 (6)	C8—C9	1.404 (7)
N1—C8	1.403 (6)	C8—C13	1.419 (8)
N2—C7	1.343 (7)	C9—C10	1.359 (8)
N2—C13	1.418 (7)	C9—H9	0.9300
N2—H2	0.8600	C10—C11	1.362 (9)
O1—C1	1.349 (6)	C10—H10	0.9300
O2—C14	1.412 (7)	C11—C12	1.342 (9)
O2—H2O	0.8771	C11—H11	0.9300
C1—C6	1.368 (8)	C12—C13	1.343 (8)
C1—C2	1.369 (8)	C12—H12	0.9300
C2—C3	1.373 (7)	C14—H14A	0.9600
C2—C7	1.486 (7)	C14—H14B	0.9600
C3—C4	1.420 (9)	C14—H14C	0.9600
			
O1—Mn1—O1^i^	180.0	C5—C4—H4	121.7
O1—Mn1—N1	88.22 (17)	C3—C4—H4	121.7
O1^i^—Mn1—N1	91.78 (17)	C6—C5—C4	120.8 (6)
O1—Mn1—N1^i^	91.78 (17)	C6—C5—H5	119.6
O1^i^—Mn1—N1^i^	88.22 (17)	C4—C5—H5	119.6
N1—Mn1—N1^i^	180.0	C5—C6—C1	122.5 (6)
O1—Mn1—O2	91.61 (17)	C5—C6—H6	118.7
O1^i^—Mn1—O2	88.39 (17)	C1—C6—H6	118.7
N1—Mn1—O2	88.72 (17)	N1—C7—N2	113.0 (5)
N1^i^—Mn1—O2	91.28 (17)	N1—C7—C2	125.5 (5)
O1—Mn1—O2^i^	88.39 (17)	N2—C7—C2	121.4 (5)
O1^i^—Mn1—O2^i^	91.61 (17)	N1—C8—C9	133.6 (5)
N1—Mn1—O2^i^	91.28 (17)	N1—C8—C13	108.5 (5)
N1^i^—Mn1—O2^i^	88.72 (17)	C9—C8—C13	117.7 (5)
O2—Mn1—O2^i^	180.0	C10—C9—C8	117.2 (5)
C7—N1—C8	106.5 (4)	C10—C9—H9	121.4
C7—N1—Mn1	123.2 (3)	C8—C9—H9	121.4
C8—N1—Mn1	129.9 (4)	C9—C10—C11	122.4 (6)
C7—N2—C13	107.6 (5)	C9—C10—H10	118.8
C7—N2—H2	126.2	C11—C10—H10	118.8
C13—N2—H2	126.2	C12—C11—C10	122.3 (6)
C1—O1—Mn1	128.6 (4)	C12—C11—H11	118.9
C14—O2—Mn1	123.6 (4)	C10—C11—H11	118.9
C14—O2—H2O	106.2	C11—C12—C13	117.2 (6)
Mn1—O2—H2O	128.8	C11—C12—H12	121.4
O1—C1—C6	117.0 (5)	C13—C12—H12	121.4
O1—C1—C2	124.1 (5)	C12—C13—N2	132.6 (6)
C6—C1—C2	118.8 (5)	C12—C13—C8	123.1 (5)
C1—C2—C3	120.2 (5)	N2—C13—C8	104.3 (5)
C1—C2—C7	120.3 (5)	O2—C14—H14A	109.5
C3—C2—C7	119.1 (5)	O2—C14—H14B	109.5
C2—C3—C4	121.0 (6)	H14A—C14—H14B	109.5
C2—C3—H3	119.5	O2—C14—H14C	109.5
C4—C3—H3	119.5	H14A—C14—H14C	109.5
C5—C4—C3	116.7 (5)	H14B—C14—H14C	109.5
			
O1—Mn1—N1—C7	26.5 (5)	C2—C1—C6—C5	−2.8 (9)
O1^i^—Mn1—N1—C7	−153.5 (5)	C8—N1—C7—N2	0.0 (6)
O2—Mn1—N1—C7	−65.2 (5)	Mn1—N1—C7—N2	173.8 (4)
O2^i^—Mn1—N1—C7	114.8 (5)	C8—N1—C7—C2	176.2 (5)
O1—Mn1—N1—C8	−161.3 (5)	Mn1—N1—C7—C2	−10.1 (8)
O1^i^—Mn1—N1—C8	18.7 (5)	C13—N2—C7—N1	0.8 (6)
O2—Mn1—N1—C8	107.1 (5)	C13—N2—C7—C2	−175.5 (5)
O2^i^—Mn1—N1—C8	−72.9 (5)	C1—C2—C7—N1	−11.6 (8)
N1—Mn1—O1—C1	−34.3 (4)	C3—C2—C7—N1	175.4 (5)
N1^i^—Mn1—O1—C1	145.7 (4)	C1—C2—C7—N2	164.2 (5)
O2—Mn1—O1—C1	54.3 (4)	C3—C2—C7—N2	−8.8 (8)
O2^i^—Mn1—O1—C1	−125.7 (4)	C7—N1—C8—C9	−176.0 (6)
O1—Mn1—O2—C14	−136.2 (5)	Mn1—N1—C8—C9	10.8 (10)
O1^i^—Mn1—O2—C14	43.8 (5)	C7—N1—C8—C13	−0.9 (6)
N1—Mn1—O2—C14	−48.1 (5)	Mn1—N1—C8—C13	−174.1 (4)
N1^i^—Mn1—O2—C14	131.9 (5)	N1—C8—C9—C10	177.3 (6)
Mn1—O1—C1—C6	−159.7 (4)	C13—C8—C9—C10	2.6 (9)
Mn1—O1—C1—C2	24.2 (8)	C8—C9—C10—C11	−1.2 (10)
O1—C1—C2—C3	178.9 (5)	C9—C10—C11—C12	0.1 (11)
C6—C1—C2—C3	2.8 (8)	C10—C11—C12—C13	−0.5 (10)
O1—C1—C2—C7	6.0 (8)	C11—C12—C13—N2	−178.6 (6)
C6—C1—C2—C7	−170.1 (5)	C11—C12—C13—C8	2.0 (10)
C1—C2—C3—C4	−1.2 (9)	C7—N2—C13—C12	179.2 (6)
C7—C2—C3—C4	171.8 (5)	C7—N2—C13—C8	−1.3 (6)
C2—C3—C4—C5	−0.5 (9)	N1—C8—C13—C12	−179.1 (5)
C3—C4—C5—C6	0.6 (10)	C9—C8—C13—C12	−3.1 (9)
C4—C5—C6—C1	1.1 (10)	N1—C8—C13—N2	1.4 (6)
O1—C1—C6—C5	−179.2 (5)	C9—C8—C13—N2	177.3 (5)

Symmetry codes: (i) −*x*+1, −*y*+2, −*z*.

### Hydrogen-bond geometry (Å, °)

**Table:**

*D*—H···*A*	*D*—H	H···*A*	*D*···*A*	*D*—H···*A*
O2—H2O···Cl1^ii^	0.88	2.25	3.107 (3)	165
N2—H2···Cl1	0.86	2.36	3.177 (5)	159

Symmetry codes: (ii) *x*, *y*+1, *z*.
